# Optical DNA Mapping of Plasmids Reveals Clonal Spread of Carbapenem-Resistant *Klebsiella pneumoniae* in a Large Thai Hospital

**DOI:** 10.3390/antibiotics10091029

**Published:** 2021-08-24

**Authors:** Sriram KK, Tsegaye Sewunet, Walaiporn Wangchinda, Teerawit Tangkoskul, Visanu Thamlikitkul, Christian G. Giske, Fredrik Westerlund

**Affiliations:** 1 Department of Biology and Biological Engineering, Chalmers University of Technology, 412 96 Gothenburg, Sweden; sriramk@chalmers.se; 2 Department of Laboratory Medicine, Karolinska Institute, 141 52 Stockholm, Sweden; tsegaye.sewunet@ki.se (T.S.); christian.giske@sll.se (C.G.G.); 3 Department of Medicine, Faculty of Medicine Siriraj Hospital, Mahidol University, Bangkok 10700, Thailand; walaiporn.wan@mahidol.ac.th (W.W.); teerawitmtmu@hotmail.com (T.T.); visanu.tha@mahidol.ac.th (V.T.); 4 Department of Clinical Microbiology, Karolinska University Hospital, 171 76 Stockholm, Sweden

**Keywords:** optical DNA mapping, plasmids, carbapenem resistance, *bla*_NDM-1_, nosocomial transmission

## Abstract

Carbapenem-resistant *Klebsiella pneumoniae* (CR-KP) in patients admitted to hospitals pose a great challenge to treatment. The genes causing resistance to carbapenems are mostly found in plasmids, mobile genetic elements that can spread easily to other bacterial strains, thus exacerbating the problem. Here, we studied 27 CR-KP isolates collected from different types of samples from 16 patients admitted to the medical ward at Siriraj Hospital in Bangkok, Thailand, using next generation sequencing (NGS) and optical DNA mapping (ODM). The majority of the isolates belonged to sequence type (ST) 16 and are described in detail herein. Using ODM, we identified the plasmid carrying the *bla*_NDM-1_ gene in the ST16 isolates and the plasmids were very similar, highlighting the possibility of using ODM of plasmids as a surrogate marker of nosocomial spread of bacteria. We also demonstrated that ODM could identify that the *bla*_CTX-M-15_ and *bla*_OXA-232_ genes in the ST16 isolates were encoded on separate plasmids from the *bla*_NDM-1_ gene and from each other. The other three isolates belonged to ST147 and each of them had distinct plasmids encoding *bla*_NDM-1_.

## 1. Introduction

Infections involving carbapenem-resistant *Klebsiella pneumoniae* (CR-KP) have become a serious problem worldwide and nosocomial infections caused by CR-KP have grown into a threatening healthcare issue in Thailand [1,2]. Broad-spectrum antibiotics, such as extended spectrum β-lactams and carbapenems, have been the first choice in treating hospital-acquired infections, but the widespread presence of extended spectrum β-lactamase- (ESBL) and carbapenemase-producing strains poses a serious threat and challenge in treating infected patients [2]. Severe misuse of antibiotics in healthcare and animal husbandry has exacerbated the problem manifold.

The World Health Organization (WHO) has classified the South East Asian (SEA) region as likely the most affected region in terms of antimicrobial resistance (AMR) [3]. KP is a common pathogen causing bacteremia, urinary tract infection and pneumonia in Thailand [4,5,6] and the prevalence of carbapenem resistance in KP has been increasing [7,8,9]. A study by Lim et al. conducted in Thailand from 2004 to 2010 showed that KP was the second most common bacterium causing bacteremia and 66% of the KP hospital-acquired bacteremia cases were caused by multidrug-resistant (MDR) isolates. The excess mortality by MDR bacteremia caused by six common bacteria was estimated to be 19,122 out of 45,209 (43%) deaths per year [1]. Although there has been a lot of attention towards AMR in recent years and surveillance studies have been conducted from time to time to understand the severity of the issue, there is still a lot of effort needed towards understanding how AMR spreads in the community and within healthcare and hospital settings [10].

New Delhi metallo-β-lactamase (NDM-1), identified for the first time in 2008 in a Swedish patient returning from India, has shown a widespread presence across the globe, including in Thailand [11,12,13]. The *bla*_NDM-1_ gene encodes the NDM-1 enzyme that confers resistance to both cephalosporins and carbapenems. Plasmids play a major role in spreading AMR, including the *bla*_NDM-1_ gene, through conjugation. The presence of multiple resistance genes in a single plasmid is common and this makes the treatment of infections challenging. Methods like S1-nuclease pulsed-field gel electrophoresis (PFGE) and PCR-based replicon typing fail to provide useful information, owing to the dynamic nature of plasmids. Next generation sequencing (NGS) has been the go-to method in recent years, where short-read sequencing still lacks accuracy in reconstructing entire plasmid sequences. This can be overcome by long-read sequencing, but this is still expensive and requires bioinformatic competency.

Optical DNA mapping (ODM) is a single molecule DNA mapping technique that relies on fluorescence imaging of single DNA molecules stretched in nanofluidic channels [14]. We have developed a protocol where one-step competitive binding labeling generates an intensity profile along each DNA molecule, based on the underlying sequence, a DNA barcode [15,16]. We have applied the method extensively to plasmids and in combination with Cas9 restriction, the gene causing the resistance can be identified [17]. The barcodes can be used to compare plasmids between different isolates and to compare with a theoretical sequence obtained from other sequencing techniques [18,19]. The ODM method has been successfully used in several studies of clinical relevance, including identifying potential conjugation during a resistance outbreak [20,21], or in patients with recurring urinary tract infections [22,23] as well as identifying clonal spread of bacteria in a hospital setting [19].

In this study, we focused on KP isolates obtained from samples collected in patients admitted to a large hospital in Thailand between 2018 and 2019. We combined short-read NGS data and ODM to investigate potential clonal spread and plasmid conjugation, focusing on plasmids carrying the *bla*_NDM-1_ gene.

## 2. Materials and Methods

### 2.1. Bacterial Isolates

Urine, fecal and sputum samples collected in 2018 and 2019 from patients admitted to Siriraj Hospital, a 2,300-bed tertiary care university hospital in Bangkok, Thailand, were subjected to a screening for carbapenem-resistant *K. pneumoniae* on CarbaID agar (bioMérieux, Marcy l’Etoile, France) and species determined with the VITEK 2 system (bioMérieux). From the screened samples that were positive for *bla*_NDM-1_, 27 isolates from 16 patients were included in the ODM study. All patients had been treated with currently used empirical antibiotics before knowing that the patient carried CR-KP isolates. Among them, nine patients had one isolate each, belonging to one of the three sample types. Five patients had two isolates, of which three patients (P6, P7 and P11) had one isolate from feces and the other from sputum collected on the same day, one patient (P15) had one isolate from feces and other from urine collected on the same day and one patient (P8) had both isolates from sputum collected three months apart. Finally, two patients (P9 and P10) had four isolates each, two from feces and two from sputum, respectively, collected at different time points. Here, isolates P9S_1 and P9F_2 were collected on the same day and isolates P9F_3 and P9S_4 were collected on the same day, three weeks later. Isolates P10S_1 and P10F_2 were collected on the same day and isolates P10F_3 and P10_S4 were collected two weeks later.

### 2.2. DNA Extraction and Whole Genome Sequencing

Two to three fresh pure colonies were taken from culture grown overnight and suspended in 200 µL suspension buffer from the EZ1^®^DNATissue Kit (QIAGEN). Then, DNA was extracted using an EZ1 Advanced DNA Bacteria Card on the EZ1 Advanced extraction system. Concentration of the extracted DNA was measured using Qubit™ 3.0 (Waltham, MA, USA). NEXTRA-XT kits were used for library preparation and sequencing was performed on an Illumina HiSeq2500 microbial sequencing platform. The raw reads were assembled using SPAdes 3.13.1 and the assembled draft genomes were used to query databases for resistance genes and virulence genes (ResFinder (https://cge.cbs.dtu.dk/services/ResFinder/ accessed on 14 February 2020) and Comprehensive Antimicrobial Resistance Database (CARD) (https://card.mcmaster.ca/analyze/rgi accessed on 14 February 2020). The draft genomes were also used for in silico prediction of capsular loci (KL) and O-lipopolysaccharide by using Kaptive/Holt lab (https://kaptive-web.erc.monash.edu/init/default/jobs accessed on 10 June 2021. Furthermore, phylogenetic relatedness of the strains was analyzed using another web tool at the Institut Pasteur: https://bigsdb.pasteur.fr/klebsiella/klebsiella.html, accessed on 10 June 2021. Tree visualization was carried out using iTOL (https://itol.embl.de accessed on 15 April 2020).

### 2.3. Plasmid Extraction

Plasmids were extracted using a NucleoBond^®^ Xtra Midi Kit (Macherey-Nagel) plasmid purification protocol. Bacterial strains were inoculated into 100 mL selective medium (Luria–Bertani with 30 µg/mL ampicillin) and incubated overnight at 37 °C, followed by DNA extraction using NucleoBond Xtra Midi Kit (Macherey-Nagel). Each isolate was pelleted by centrifugation at 7000 rpm, for 10 min at 4 °C. The pellet was resuspended in resuspension buffer, lysed, and purified on columns according to the manufacturer’s recommendations. The eluted plasmid DNA was precipitated with isopropanol and washed once with 70% ethanol and dried at ambient temperature. The dried pellet was reconstituted in 100 μL TE buffer. The DNA concentration was measured using a Qubit™ dsDNA BR Assay Kit (ThermoFisher).

### 2.4. Optical DNA Mapping (ODM)

**Figure 1:**
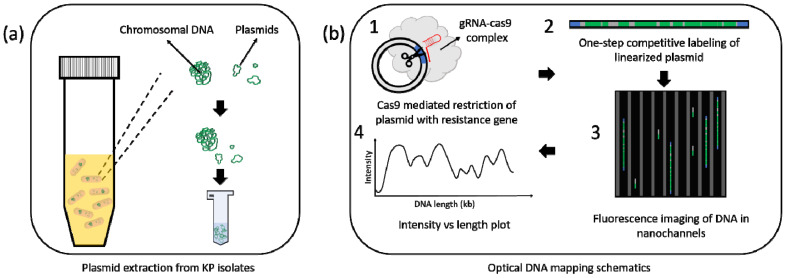
ODM schematics. (**a**) Plasmids extracted from bacteria collected from patients (Materials and Methods). (**b**) 1. Restriction of plasmids with Cas9, targeting bla_NDM-1_, *bla*_CTX-M-15_ or *bla*_OXA-232_ gene. 2. One-step competitive labeling of plasmids using YOYO-1: Netropsin. 3. Imaging of stretched DNA molecules in nanofluidic devices. 4. Image analysis to obtain barcodes or intensity profiles of DNA molecules.

The ODM assay has been well established in our earlier studies and is shown schematically in Figure 1 [14,17,19,21,22]. Custom-made crRNAs targeting *bla*_NDM-1_ (5′ CCGCTGCATTGATGCTGAGC 3′), *bla*_CTX-M-15_ (5′ CCGTCGCGATGTATTAGCGT 3′) and *bla*_OXA-232_ (5′ TGGAATGAGAATAAGCAGCA 3′) genes, respectively, were used. 

DNA stretching for ODM was performed using a micro–nanofluidic device, consisting of 120 parallel nanochannels of dimensions 100 nm (height), 150 nm (width) and 500 µm (length). Images were obtained using an inverted fluorescence microscope (Zeiss AxioObserver.Z1) with a 100X oil objective (Zeiss, 1.46 N.A.), FITC filter (488 nm excitation and 530/50 nm emission) and a Photometrix Evolve sCMOS camera. A nanofluidic device with parallel nanochannels allowed us to include tens of DNA molecules in each image (20 frames, 100 ms exposure) and a total of 15 to 20 images were collected. This process takes 20 to 30 min, thus allowing imaging of multiple samples in one day.

The images were processed using a custom-made Matlab code. The details of the image analysis workflow have been described in detail elsewhere [17,18]. Kymographs of linear molecules were sorted based on their lengths. Each length group (if there were more than one) was then checked to see if the cluster of molecules in the group has the cut at a specific region (presence of *bla*_NDM-1_ gene) or at random sites due to light- or shear-induced breaking (absence of the gene). The length of the molecules in the Cas9 cut cluster was estimated using λ-DNA (48,502 bp, New England Biolabs) as a reference and are presented in units of kilobases (kb) [24].

The average intensity profile (barcode) from each isolate was then compared against other isolates to find if the compared barcodes matched completely, partially or were entirely different. Details on the statistical framework for ODM analysis have been described in detail in our earlier works [15,19,25]. The similarity between isolates was indexed using *p*-values [19,21]. For a complete match (denoted as *p_comp_*), barcodes of similar lengths (within 10% length difference) were stretched to the same length. If *p_comp_* ≤ 0.01 and the gene location was the same, then the barcodes compared were classified as identical. Next, the barcodes were compared using their real length (significance denoted as *p_part_*) obtained from our measurements. Here, the barcode of the shorter molecule was moved along the barcode of the longer molecule to find the best match. If *p_part_* ≤ 0.01, they matched partially due to an insertion or deletion in one of the two compared plasmids. If both *p*-values were > 0.01 or if the gene location was different from other plasmids in comparison, the plasmids were classified as different.

## 3. Results

KP isolates resistant to commonly used antibiotics were collected at the medical wards at Siriraj Hospital in Bangkok, Thailand in 2018 and 2019. Short-read NGS was used to characterize a set of KP isolates consisting of a total of 27 isolates from 16 patients. The isolates were named with a patient number, the sample type and, if more than one isolate was collected from a patient, they were listed in numerical order. For example, P9F_2 represents the isolate from patient 9, sample type is feces, and it is the second isolate from patient 9. Details of all the isolates involved in this study are listed in Table 1.

**Figure 2:**
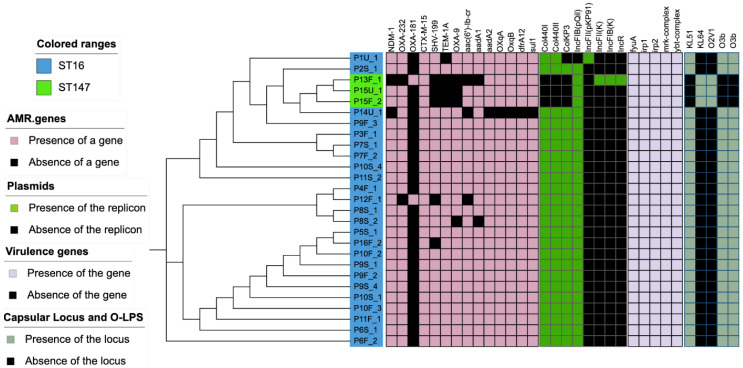
Heat map showing sequence type, resistance genes, plasmid replicons encoded, virulence genes, capsular types and O-lipopolysaccharide locus.

AMR genes were detected using the assembled draft genome blasted to ResFinder at the Center for Genomic Epidemiology and the Comprehensive Antimicrobial Resistance Database (CARD). Multilocus sequence type (MLST) was determined using microSALT, an in-house bioinformatic tool at the Department of Clinical Genomics, SciLifeLab (Stockholm, Sweden). Most of the strains were identified as ST16 (n = 24) and ST147 (n = 3). All of them encoded resistance genes for carbapenemases, ESBLs, aminoglycosides, fluoroquinolones, tetracyclines and sulfonamides. All of the ST16 strains showed a similar resistome profile that included carbapenemases (*bla*_NDM-1_ and *bla*_OXA-232_), ESBLs and other lactamases (*bla*_CTX-M-15_, *bla*_SHV-199_, *bla*_TEM-1A_ and *bla*_OXA-9_), aminoglycosides (aac(6’)-Ib, aadA1 and aadA2), fluoroquinolones (oqxA, and oqxB), trimethoprim (dfrA12) and sulfonamides (sul1). All strains in this study showed similar virulome profiles with virulence genes involved in iron uptake and/or metabolism (fyuA, irp1, irp2, ybtQ) and type-3 fimbriae (mrkA complex) were encoded by both the ST147 and ST16 strains. However, the two sequence types differed in plasmidome, capsular type, and O-LPS profile. ST16 encoded for KL-51 capsular type and O3b O-LPS, whereas ST147 encoded for KL-64 capsular type and O2v1 O-LPS (Figure 2).

**Figure 3:**
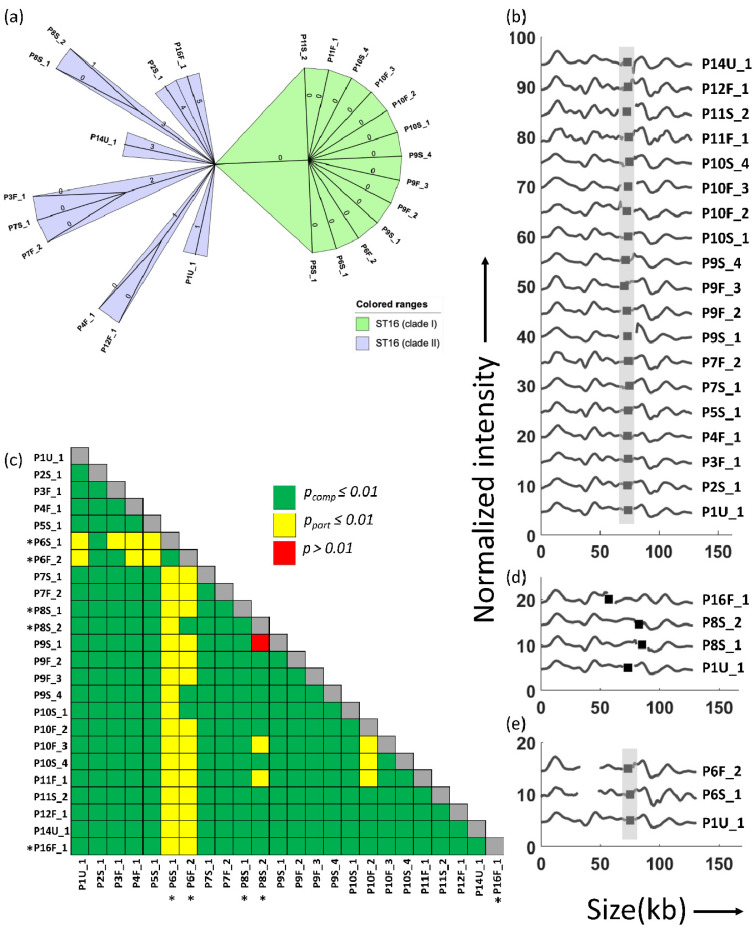
(**a**) Minimum spanning tree (grape tree) of all ST16 isolates based on cgMLST. The numbers on the solid black lines correspond to the number of alleles that differ between each isolate. (**b**) Barcodes of identical plasmids from ST16 isolates, stretched to 128 kb. The x-axis shows the length of the plasmids in kb and the y-axis is the normalized intensity in arbitrary units, where the intensity plot of each isolate is shifted five units vertically for clarity. (**c**) Similarity matrix of the plasmids from the ST16 isolates. Identical barcodes when stretched using the same size of 128 kb are considered as complete matches (*p_comp_* ≤ 0.01) and are labeled green; identical barcodes when stretched using actual sizes measured by the ODM experiments are considered as partial matches (*p_part_* ≤ 0.01) and are labeled yellow; *p* > 0.01 when stretched using similar lengths or measured lengths are considered “no match” are labeled red. Isolates with plasmids different from the common ST16 plasmid in 3b are denoted by a *. (**d**) ST16 isolates with barcodes identical to the common isolates, but with shifted gene site. P8S_1 and P8S_2 (*p_comp_* ≤ 0.01) with ~10 kb shift in gene site in comparison to P1U_1 and P16F_1 with ~15 kb shift in gene site compared to P1U_1. (**e**) Isolates P6S_1 and P6F_2 with plasmids shorter by 12 kb and 7 kb compared to the average length of the common ST16 plasmids. Black squares and gray shades represent the location of the *bla*_NDM-1_ gene in (**b**,**d**,**e**).

It is evident from the spanning tree diagram obtained from NGS data that all the ST16 isolates are very closely related (Figure 3a). ST16 cluster 1 has 13 isolates that are highly identical with no difference in any alleles. The remaining ST16 isolates fall in cluster 2 with a difference of one to five alleles, which is still rather small.

Of particular interest in this study were the plasmids and we used ODM to characterize them. By combining ODM with Cas9 targeting the *bla*_NDM-1_ gene, a plasmid carrying *bla*_NDM-1_ was identified in all the 27 isolates studied. The length measurements that are part of the ODM analysis showed that the plasmid carrying *bla*_NDM-1_ was ~128 kb in all 24 ST16 isolates. In the next step, we therefore compared the barcodes for all these isolates when stretching them to the same length (Figure 3b). We found that the barcodes of plasmids carrying the *bla*_NDM-1_ gene were similar in 19 out of 24 of the ST16 isolates (*p_comp_* ≤ 0.01 and identical *bla*_NDM-1_ gene location, see Materials and Methods). Figure 3c shows a similarity matrix, where green boxes represent *p_comp_* ≤ 0.01, yellow boxes represent *p_part_* ≤ 0.01 and red boxes represent *p* > 0.01.

The *bla*_NDM-1_-encoding plasmid in the other isolates differed in several different ways. P8S_1 and P8S_2 had a similar barcode to the common ST16 plasmid (*p_comp_* ≤ 0.01), but the *bla*_NDM-1_ gene was shifted by ~10 kb compared to the common gene location (Figure 3d). P16F_1 also had a plasmid similar to the common plasmid (*p_comp_* ≤ 0.01), but had the *bla*_NDM-1_ gene shifted by ~15 kb in the other direction (Figure 3d). Translocation of genes may influence transcription and expression of genes. It was beyond the scope of the study to investigate this further, as it is already well known that the carbapenemase gene confers phenotypic resistance to carbapenems, which was also found in this case. Thus, there is reason to believe that the carbapenemase gene remained fully functional. Furthermore, NGS showed that the *bla*_NDM-1_ gene had a 100% matching identity in sequence to the reference gene. P6S_1 (114 kb) and P6F_2 (121 kb) showed better matches to the common plasmid when using their measured lengths (*p_part_* ≤ 0.01), indicating that they were ~12 kb and ~7 kb shorter than the average length of 128 kb (Figure 3e). The *bla*_NDM-1_ gene sites in P6S_1 and P6S_2 were identical to the common ST16 plasmid.

We have recently demonstrated how ODM can be a potential tool to identify the clonal spread of bacteria carrying the *bla*_CTX-M-15_ gene [21]. As a next step, we therefore compared the ODM data for the *bla*_NDM-1_ encoding plasmid with the cgMLST data, focusing on the ST16 strains. The ODM data and cgMLST data show an excellent overlap and demonstrate that the strains with a very similar plasmid also had a very similar core genome. An interesting observation upon comparing ODM and cgMLST data is that the three isolates P8S_1, P8S_2 and P16F_1 with a shifted gene location were also among the isolates with higher allelic differences, three, four and five alleles, respectively, compared to the common ST16 cluster.

**Figure 4:**
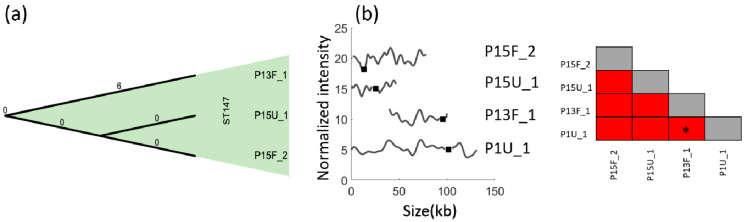
(**a**) Minimum spanning tree (grape tree) for all ST147 isolates based on cgMLST. The numbers on the solid black lines correspond to the number of alleles that differ between each isolate. (**b**) Barcodes of all ST147 isolates compared with the common ST16 plasmid (P1U_1) where the x-axis shows the length of plasmids in kb and the y-axis is the normalized intensity in arbitrary units, and the intensity plot of each isolate is shifted five units vertically for clarity. Black squares and gray shades represent the location of the *bla*_NDM-1_ gene. To the far right is the similarity matrix of *bla*_NDM-1_-carrying plasmids in these three ST147 isolates. P13F_1 vs. P1U_1 yielded identical barcodes for overlapping regions, but with *p* > 0.01, and is denoted by a * in the similarity matrix.

When we investigated the cgMLST data for the three ST147 isolates, all of them showed high similarity with no allelic difference (Figure 4a). On the contrary, ODM results indicate that the plasmids are all different from each other and from the common ST16 isolates (Figure 4b). P13F_1, P15U_1 and P15F_2 are the three ST147 isolates, and they all had unique plasmid barcodes (*p* > 0.01, Figure 4b). Isolate P13F_1 had a 55 kb plasmid with the *bla*_NDM-1_ gene and, interestingly, this plasmid had a barcode identical (*p* = 0.02) to the common ST16 barcode for the overlapping regions and the gene location was similar too. The *p*-value was slightly higher than 0.01 owing to the large difference in lengths. For patient P15, we had two isolates, both belonging to ST147. P15U_1 had the *bla*_NDM-1_ gene in a 50 kb plasmid while P15F_2 had the *bla*_NDM-1_ gene in an 85 kb plasmid. A comparison between the three ST147 isolates showed that the plasmids were of different lengths and the gene locations were different.

**Figure 5:**
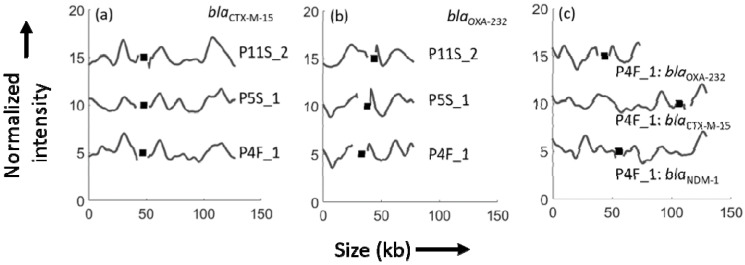
Barcodes of plasmids from three ST16 isolates for (**a**) the *bla*_CTX-M-15_ gene. All three isolates show the *bla*_CTX-M-15_ gene in a ~128 kb plasmid and the genes (black squares) are in the same location for all three cases. (**b**) The *bla*_OXA-232_ gene in the same three isolates showing that the gene is in the same location (black squares) in a plasmid of length ~75 kb. (**c**) Comparison of barcodes showing all three genes, *bla*_NDM-1_, *bla*_CTX-M-15_ and *bla*_OXA-232_, (black squares) in isolate P4F_1. The x-axis shows the length of plasmids in kb and the y-axis is the normalized intensity in arbitrary units, where the intensity plot of each isolate is shifted five units vertically for clarity.

An important feature of the ODM methodology is the possibility to assign resistance genes to specific plasmids. During the ODM analysis targeting the *bla*_NDM-1_ gene, we noticed many plasmids that were not cut by Cas9, suggesting that the samples contained several different plasmids. We were therefore interested in investigating on which plasmid the *bla*_OXA-232_ and *bla*_CTX-M-15_ genes were located in three representative ST16 isolates. ODM targeting the *bla*_CTX-M-15_ gene on isolates P4F_1, P5S_1 and P11S_2 showed that they were all present in an identical 128 kb plasmid (Figure 5a). ODM targeting the *bla*_OXA-232_ gene in the same three isolates (P4F_1, P5S_1 and P11S_2) showed that the gene was on an identical 75 kb plasmid (Figure 5b). Interestingly, the barcodes of the plasmid with the *bla*_CTX-M-15_ gene and the *bla*_NDM-1_ gene were different (*p* > 0.01, Figure 5c), suggesting that this isolate had two different plasmids of similar size, that would be indistinguishable with a method that only measures plasmid size. The 75 kb plasmid carrying *bla*_OXA-232_ did not show any similarity to either of the two larger plasmids (Figure 5c).

## 4. Discussion

The goal of the project was to analyze bacterial isolates resistant to carbapenems collected at a large hospital in Bangkok, Thailand with particular focus on the plasmids of the isolates. We combined NGS and ODM to identify possible clonal spread of bacteria in the hospital.

The vast majority of the isolates belonged to ST16 and cgMLST confirmed that they were highly similar. The strains belonging to this clone featured double carbapenemases, *bla*_NDM-1_ and *bla*_OXA-232_, as well as *bla*_CTX-M-15_. Plasmid-mediated genes encoding resistance to fluoroquinolones, aminoglycosides, trimethoprim and sulfonamides were also found, resulting in a multidrug-resistant phenotype. ST16 with a combination of NDM-1 and OXA-232 has previously been described in Italy [26]. This clone is known as a successful clone that can also disseminate KPC [27]. The ST16 strain was of K-type 51 and featured virulence genes related to iron uptake and metabolism, as well as type-3 fimbriae.

ODM analysis of the ST16 strains demonstrated that the same plasmid encoding *bla*_NDM-1_ was present in 19 of the isolates. In the other five, the plasmid encoding *bla*_NDM-1_ was similar but with either a slightly different location of the *bla*_NDM-1_ gene, or a significant difference in plasmid length. The combination of NGS and ODM data gives a strong indication of a nosocomial spread of this strain in the hospital, since both methods show, in complementary ways, that the isolates are highly similar. An alternative explanation for the high prevalence of a bacterial strain with an identical plasmid found among the patients studied could be that this strain is abundant in the community, but given the nature of some KP clones as primarily nosocomial pathogens, this is a less likely explanation. Both scenarios are alarming and emphasize the frequent need for studies like this to understand the spread of resistance genes, which in turn will help the fight against antibiotic resistance.

We recently demonstrated that it is possible to identify clonal spread of bacteria by identifying bacterial plasmids using ODM. That study was carried out for *E. coli* and *bla*_CTX-M-15_, and we show here that it is also possible for KP and *bla*_NDM-1_ and *bla*_OXA-232_, indicating that the method has general utility. The ODM can be performed on its own, which might be interesting in lower-resource settings where sequencing might not be feasible, but imaging using a simple microscope can be carried out.

An important feature of ODM is its capability to, in contrast to for example PCR, determine on which plasmid in a sample a certain gene is present. This is of interest from an epidemiological perspective, since if several genes are located on the same plasmid, they will conjugate together, and a single conjugation event can lead to an MDR recipient bacterium. In this study, we confirmed, using ODM, that the *bla*_NDM-1_, *bla*_OXA-232_ and *bla*_CTX-M-15_ genes were on three different plasmids.

Interestingly, two of the plasmids were of very similar length and it would not have been possible to discriminate them using a method, such as PFGE, that only determines plasmid lengths. ODM, on the other hand, readily confirmed that the two plasmids had very different barcodes. In addition, the results further strengthen the discussion above on the relatedness of the strains since not only was one plasmid similar between the strains, but there were three similar plasmids in total.

ODM also allowed us to confirm that the other isolates analyzed in the outbreak (belonging to ST147) were completely different, also regarding plasmids. This confirms that conjugation did not occur between these two bacterial strains. More importantly, the plasmids were also different between the three isolates, even if two of them were collected from the same patient and they all had a very similar cgMLST. This could indicate that ST147 is a common clone in the community setting, but more isolates would be needed to confirm this.

## 5. Conclusions

In conclusion, we demonstrated that ODM of plasmids is a suitable technique to identify nosocomial transmission of bacteria. It complements NGS by characterizing plasmids in detail and the analysis confirms that most cases of *bla*_NDM-1_ in this study were caused by an identical strain belonging to ST16.

## Figures and Tables

**Table 1:** Information of K. pneumoniae isolates used in this study. Column 1 shows the isolate code (U = Urine, S = Sputum, F = Feces), column 2 provides information on the source from which the sample was obtained, column 3 shows the multilocus sequence type (MLST) of each isolate, column 4 shows the length of the plasmid (in kb) carrying the *bla*_NDM-1_ gene in each isolate studied using ODM, column 5 shows the length of the plasmid (in kb) carrying the *bla*_CTX-M-15_ gene in isolates studied using ODM and column 6 shows the length of the plasmid (in kb) carrying the *bla*_OXA-232_ gene in isolates studied using ODM.

Isolate Code	Sample Type	MLST Type	Plasmid with *bla*_NDM-1_ Gene (Kb)	Plasmid with *bla*_CTX-M-15_ Gene (kb)	Plasmid with OXA-232 Gene (kb)
P1U_1	Urine	16	131		
P2S_1	Sputum	16	136		
P3F_1	Feces	16	125		
P4F_1	Feces	16	130	127	76
P5S_1	Sputum	16	128	128	72
P6S_1	Sputum	16	114		
P6F_2	Feces	16	121		
P7S_1	Sputum	16	130		
P7F_2	Feces	16	124		
P8S_1	Sputum	16	134		
P8S_2	Sputum	16	122		
P9S_1	Sputum	16	129		
P9F_2	Feces	16	128		
P9F_3	Feces	16	125		
P9S_4	Sputum	16	123		
P10S_1	Sputum	16	130		
P10F_2	Feces	16	126		
P10F_3	Feces	16	130		
P10S_4	Sputum	16	131		
P11F_1	Feces	16	138		
P11S_2	Sputum	16	132	128	77
P12F_1	Feces	16	130		
P13F_1	Feces	147	55		
P14U_1	Urine	16	124		
P15U_1	Urine	147	52		
P15F_2	Feces	147	85		
P16F_1	Feces	16	122		

## Data Availability

All data can be obtained from the corresponding authors upon reasonable request.
